# A gene co-expression network model identifies yield-related vicinity networks in *Jatropha curcas* shoot system

**DOI:** 10.1038/s41598-018-27493-z

**Published:** 2018-06-15

**Authors:** Nisha Govender, Siju Senan, Zeti-Azura Mohamed-Hussein, Ratnam Wickneswari

**Affiliations:** 1 School of Environmental and Natural Resource Sciences, Faculty of Science and Technology, Universiti Kebangsaan Malaysia, 43600 UKM Bangi, Selangor Malaysia; 2 Center for Bioinformatics Research, Institute of Systems Biology (INBIOSIS), Universiti Kebangsaan Malaysia, 43600 UKM Bangi, Selangor Malaysia; 3 School of Biosciences and Biotechnology, Faculty of Science and Technology, Universiti Kebangsaan Malaysia, 43600 UKM Bangi, Selangor Malaysia

## Abstract

The plant shoot system consists of reproductive organs such as inflorescences, buds and fruits, and the vegetative leaves and stems. In this study, the reproductive part of the *Jatropha curcas* shoot system, which includes the aerial shoots, shoots bearing the inflorescence and inflorescence were investigated in regard to gene-to-gene interactions underpinning yield-related biological processes. An RNA-seq based sequencing of shoot tissues performed on an Illumina HiSeq. 2500 platform generated 18 transcriptomes. Using the reference genome-based mapping approach, a total of 64 361 genes was identified in all samples and the data was annotated against the non-redundant database by the BLAST2GO Pro. Suite. After removing the outlier genes and samples, a total of 12 734 genes across 17 samples were subjected to gene co-expression network construction using petal, an R library. A gene co-expression network model built with scale-free and small-world properties extracted four vicinity networks (VNs) with putative involvement in yield-related biological processes as follow; heat stress tolerance, floral and shoot meristem differentiation, biosynthesis of chlorophyll molecules and laticifers, cell wall metabolism and epigenetic regulations. Our VNs revealed putative key players that could be adapted in breeding strategies for *J. curcas* shoot system improvements.

## Introduction

*Jatropha curcas* L. or the physic nut is an environmentally friendly and cost-effective feedstock for sustainable biofuel production. The tree-like shrub has a productive life span of 50 years and endows oil-rich (37–50%) seeds^1–3^. The *Jatropha* crude oil (JCO) physiochemical characteristics are highly desired for biodiesel production; long chain fatty acids, low acidity, low viscosity, good stability and a relatively high cetane number. In addition, JCO-based biodiesel is able to perform better than the conventional petro-diesel in terms of engine maintenance and carbon dioxide emission^4,5^. Edible vegetable oils are primary candidates for biofuel feedstock. Developed nations such as the US, UK and Canada are dependent on soybean, canola, sunflower, rapeseed and corn oil for biodiesel feedstock while others such as Malaysia and Indonesia have deployed the palm oil. The usage of edible vegetable oil for biofuel is feasible provided a surplus supply which exceeds the consumption demand is met^6–8^. Under this scenario, the JCO also a toxic non-edible oil with lucrative properties for biofuel production, could potentially resolve the present food versus fuel pressure faced by the edible vegetable oils. At present, the commercialization of *Jatropha*-based biofuel is hampered by a lack in planting materials with consistent flowering, fruiting and seed yields throughout the planting cycles.

In higher plants, the post-embryonic developments of vegetative and reproductive tissues are inter-dependent^9,10^. Yield component is affected by reproductive structures such as seed, flower and axillary meristem and the reproductive phase is only reached after a substantial period in vegetative phase. Nevertheless, both the vegetative and reproductive phase remain confounded to one another for the reproductive success in plants. In agronomic evaluation of *J. curcas*, numerous studies have shown strong association between *J. curcas* yield and reproductive structures such as flower number, pollen fertility, male to female flower ratio and seed number^1,11,12^. In addition, breeding and management programs had utilized extensive number of genomics and molecular approaches for the examination of *J. curcas* reproductive structures^13–20^. In a phenotypic evaluation of *J. curcas* accessions from South-East Asia, the yield-associated traits showed positive correlation to number of branches^21^. Likewise, in another study conducted in India, the seed yield of 2-year-old *J. curcas* showed significant correlation to number of secondary and tertiary branches per plant^12^. Despite interesting agronomic findings which had indicated strong association between the shoot system and yield, the molecular studies of *J. curcas* shoot system is under-emphasized for yield enhancement breeding strategies.

The aerial shoot and its small population of mitotically dividing cells at the center, called the shoot apical meristem (SAM) are critical tissues required to start the vegetative and subsequent reproductive development in plants. The SAM maintains a pool of undifferentiated cells in the center while generating above-ground plant organs: the stems, leaves and flowers. The reiterative formation of lateral organs and basal regions from the SAM requires communication among cells within SAM and between the SAM and incipient organs^22–24^. The indeterminate shoot growth (vegetative phase) is terminated by the determinate growth of flowers (reproductive phase) when SAM switches from inflorescence meristem into floral meristem, and the entire developmental phase changes are orchestrated by a complex gene network^24^. Gene expression patterns obtained from the aerial shoot, together with shoot bearing inflorescence (basal region) and inflorescence provide essential information to elucidate gene associations among the reproductive-related shoot tissues. Therefore, a gene co-expression network which predicts potential functional relationship between genes and subsequently predicts a gene’s function^25,26^ is employed to investigate the gene-to-gene relationship in *J. curcas* reproductive-related shoot system.

Biological networks are widely used to characterize the interactions among genes in order to understand the biological processes and functions underpinning traits of interest. Genes with similar expression patterns are likely to have similar functions, which may have been regulated by the same mechanism or a joint transcription factor^27,28^. Expression data generated from transcriptomes are primarily used to build networks. High-throughput, genome sequencing technology offers an array of techniques for the measurement of gene expression specific to a given experimental condition, tissue or developmental stage. Gene co-expression is the expression profiles of a gene across the different conditions and that they are compared against other genes to acquire similar relationships between the genes. In a network, nodes represent genes and the interconnecting edges between the nodes reflect the degree of correlated expressions. A subset of nodes that are tightly connected to each other is a module. Within a module, the highly connected genes, also referred as ‘hub genes’ are likely to have important biological function^25,26,29^. A gene co-expression network is widely used to infer the ‘true’ biological process which includes interactions and other combined activities at the cellular system.

In a previous paper^30^, we identified differentially expressed (DE) genes in *J. curcas* inflorescence relative to shoot tissues and categorized them according to their ontologies term. A differential gene expression analysis scores for statistically significant DE genes between a pair of treatments in accordance to the absolute count value. Valuable information on relationship among the DEs (Guilt-by-Association) remains disentangled, despite their significant importance in gene function prediction and regulatory mechanisms. Thus, we constructed a gene co-expression network using petal, a publically available R library. The RNA-seq based transcriptomes generated from *J. curcas* shoot system were fed into petal to obtain a biologically meaningful gene co-expression network model which follows the scale-free and small-world properties. A list of DE genes^30^ with yield-related terms (biological process) were selected to construct vicinity networks (VNs). Our VNs containing groups of functionally annotated genes underlying yield-related biological processes in *J. curcas* such as flowering, epigenetics, stress tolerance and the biosynthesis of chlorophyll molecules and laticifiers are putatively characterized based on literature.

## Material and Methods

### Isolation of rna, assembly construction and transcriptome analysis

For RNA-sequencing library preparation, mature shoots subtending an inflorescence at 2.5 cm from the base of peduncle, aerial shoots at 2.5 cm from the tip of the apex and whole inflorescences were collected from six *Jatropha* plants. Total RNA was isolated using a combined modified method; CTAB + silica column^31^ and RNeasy Plant mini kit (Qiagen, Hilden, Germany) and the concentration was measured using a Nanodrop ND-10000 spectrophotometer (NanoDrop Technologies, USA). The quality and integrity of total RNA were determined at 260/280 nm ratio by NanoBioanalyzerRNA-6000 analysis (Agilent-Technologies). The cDNA library was constructed according to the SureSelect Strand-Specific RNA Library Prep for NGS Workflow protocol (Agilent Technologies, USA) and the corresponding fragmented genomic DNA was sequenced using Illumina HiSeq. 2500 (Yourgene Bioscience, Taiwan). The output, raw fastq reads were trimmed using the Trim Galore package and aligned to *J. curcas* genomic sequences obtained from Kazusa’s Jatropha Genome Database version r4.5 (http://ftp.kazusa.or.jp/pub/jatropha/). Alignment was performed using the STAR aligner (https://github.com/alexdobin/STAR) and Cufflinks were used to produce a merged assembly. Assembly construction was performed using the CLC Genomics Workbench (CLC bio, USA). For a descriptive characterization, the *J. curcas* transcripts were annotated using BLAST2GO PRO 4.0 suite^32,33^; BLAST, mapping, InterPro Scan and annotation.

### Data: Quality control and detection for outliers

The transcripts were normalized into counts per million (cpm) and lowly expressed genes were filtered out as follows: retain genes that are expressed at cpm >5 in all samples. The shoot and inflorescence treatments were clustered to detect presence/absence of outliers. A cluster of dendogram for each group of samples was generated using the Jaccard distance (vegan package). To confirm dissimilarities between the treatment groups, a multidimensional (MDS) plot was constructed using limma and edgeR packages.

### Gene co-expression network algorithm and parameters

The gene co-expression network was constructed according to petal, a novel co-expression modeling system^25^. This system is designed for construction of biologically meaningful networks with a small-world and scale free structure. Under this approach, Spearman (SP) was selected to generate an association matrix. The SP applies for transcriptome count data with a non-normal distribution (Supp. 1). The association matrix was converted to adjacency matrices using a series of thresholds generated by petal’s default function (Add. 2) and the “best threshold” was selected after a customized refining-threshold procedure. Criteria for the network construction were as follows: Coefficient of determination (R^2^) >0.9, average cluster coefficient (meanCC) <0.5, meanPath <5, average path length: percentage of original dataset utilized in the network model (%used) >95, and percentage of vertices that are in the biggest component of the network model (%bigComp) >95. From the gene co-expression network (network model), we extracted vicinity networks (VNs) and expression profiles of samples using nine yield-related genes, previously described as differentially expressed genes in inflorescence^30^. The gene co-expression network and VNs were drawn, visualized and annotated using the Cytoscape 3.0 software. Topological measures were generated using Network analyzer^34^.

### Pathway analysis

Each vicinity network (VN) was subjected to pathway enrichment analysis using the KaPPA-View4 Jatropha (http://kpv.kazusa.or.jp/kpv4) metabolic pathway database^35^. All genes in each VN were categorized according to KEGG pathways as follow: metabolism, genetic information processing, environmental information processing and cellular process. Correlations (>0.9) of gene to gene co-expression are indicated as red curves and each gene is represented as square box on the pathway maps.

### Data availability

The transcriptome sequence data utilized in this study is found in National Center for Biotechnology (NCBI)’s Sequence Read Archive (SRA) database with the accession number, SRP090662 (https://www.ncbi.nlm.nih.gov/sra/?term=SRP090662).

## Results and Discussion

### Transcriptome analysis

The number of clean and mapped reads obtained for 18 RNA libraries sequenced, the descriptive statistics of the transcriptomes and the sample descriptions are presented in Table 1. The number and percentage of reads mapped onto genomic loci and multiple loci ranged from 81.02–88.22 and 8.15–11.25, respectively. After removal of low-quality reads, the total number of reads in the 18 samples ranged from 22 961 194–35 876 223. Reads were mapped onto *J. curcas* reference genome and a total of 64 361 transcript sequences were obtained from the 18 transcriptomes. The average assembly size for each transcriptome ranged from 16 886 160 to 26 833 093 transcripts. From the 21 188 sequences with BLASTX hits, only 5163 and 32 299 sequences acquired mapping and annotation respectively. The InterPro Scan (IPS) revealed 34 438 sequences without an IPS match, while 29 923 and 16 615 sequences scored match with IPS and with Gene Ontology (GO), respectively. The transcripts with GO terms showed 673 ECs that are associated with 102 KEGG pathways. Sequence search against the Rfam database indicated presence of 82 non-coding RNAs (Table 1).

**Table 1:** The *Jatropha curcas* shoot system transcriptome data.

Attributes	Values	
Read length	2 × 100 bp	
Sequence analyzed (pair)	22 961 194–35 876 223	
Filtered	3.49–4.75%	
Aligner reads (pair) input	22 053 488–34 343 944	
Uniquely mapped reads	81.02–88.22%	
Reads mapped to multiple loci	8.15–11.25%	
Shoot Biosample ID	SAMN05827448	SAMN05827459
	SAMN05827450	SAMN05827460
	SAMN05827452	SAMN05827462
	SAMN05827454	SAMN05827463
	SAMN05827455	SAMN05827464
	SAMN05827456	
	SAMN05827458	
Inflorescence Biosample ID	SAMN05827449	SAMN05827457
	SAMN05827451	SAMN05827461
	SAMN05827453	SAMN05827465
Reads mapped to gene model	16 886 160–26 833 093	
With BLASTX hits	21 188	
With mapping	5163	
With GO annotation	32 299	
With IPS	29 923	
IPS with GOs	16 615	
Non-coding RNAs (RFAM)	82	
KEGG pathways	102	

### Detection for outliers within and between the treatments

**Figure 1:**
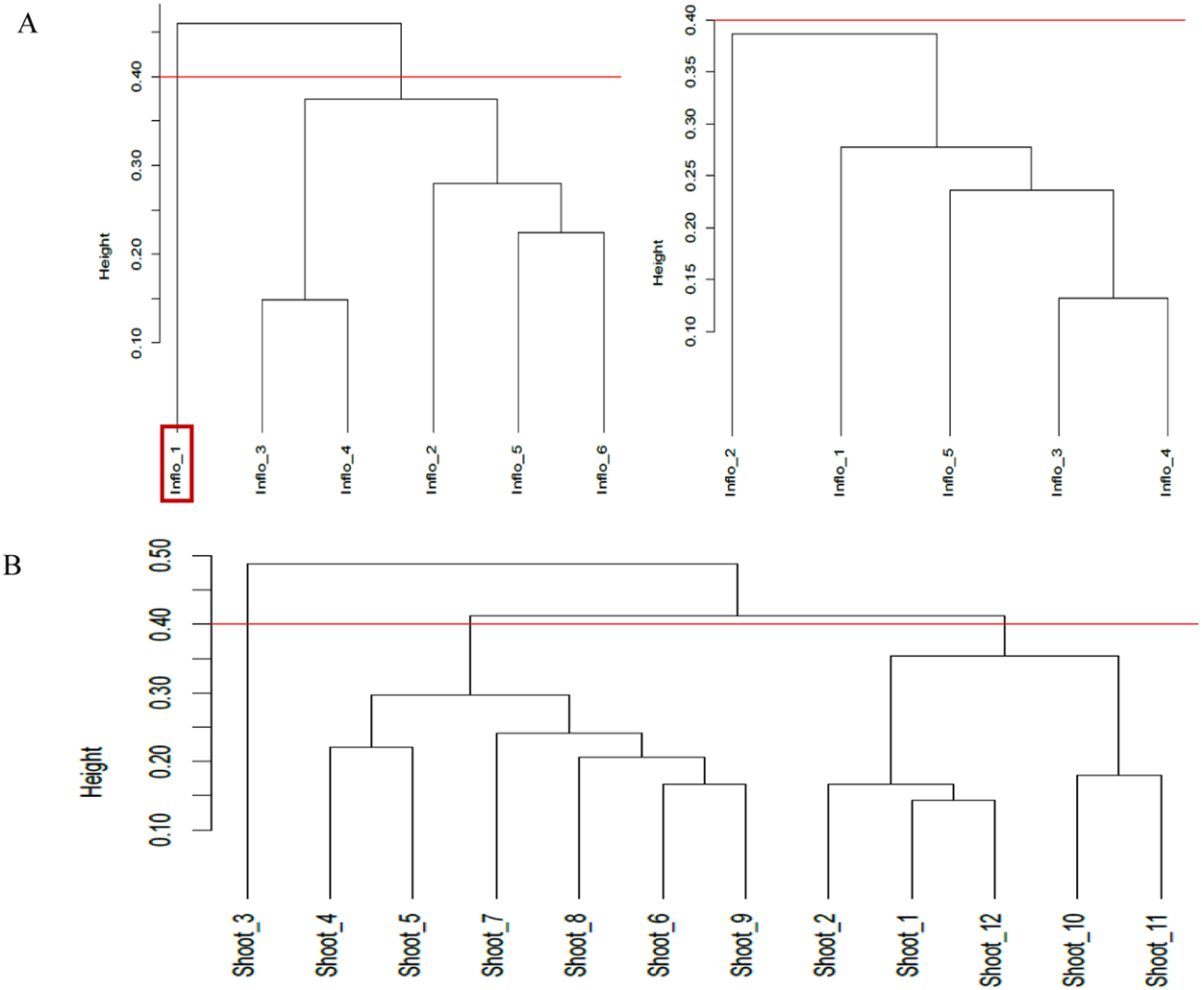
Jaccard-based similarity dendogram applied to *Jatropha curcas* inflorescence (**A**) and shoot (**B**) transcriptome data. Read line indicates cut-off height at 0.40. (A) Cluster of dendogram obtained before (left) and after (right) sample editing; the original data set of six samples (left) indicates presence of an outlier (boxed).

**Figure 2:**
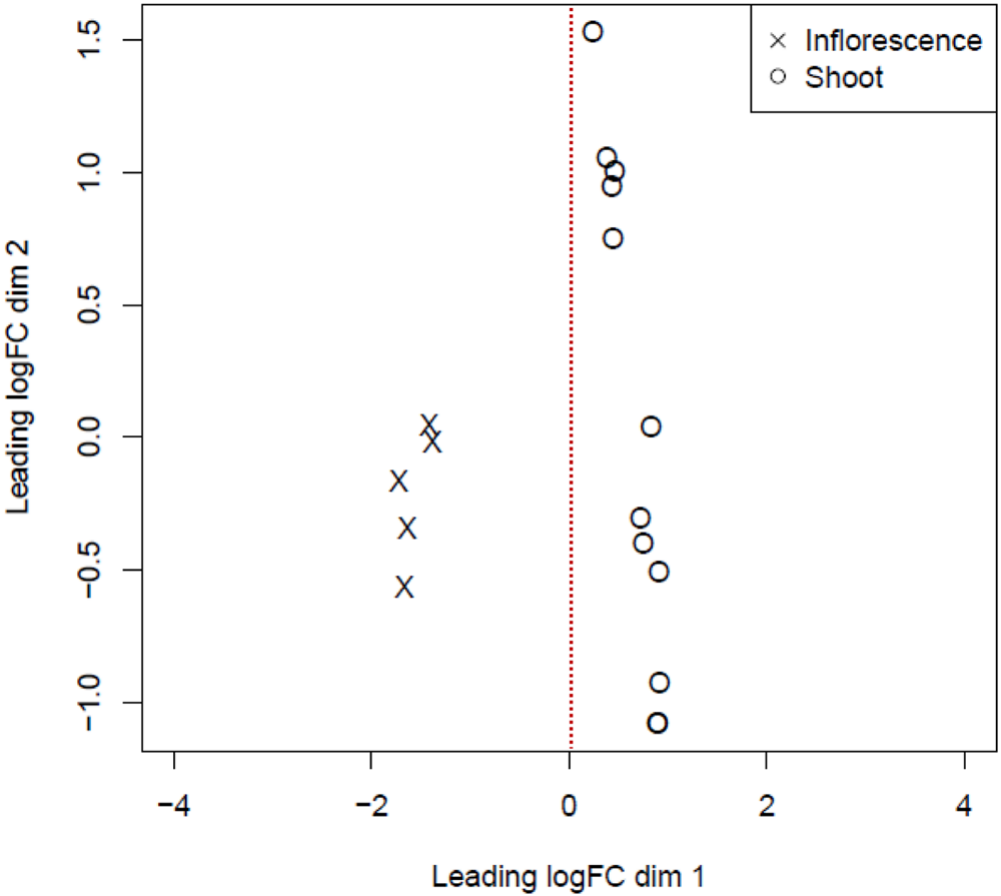
A multidimensional scaling (MDS) plot shows the dissimilarities between the *Jatropha curcas* shoot and inflorescence samples at dimension (dim) 1 with leading logFC = 0. Twelve circles (shoot) scattered at the right panel separates the five crosses (inflorescence) clustered on the left panel from the logFC dim 1 = 0 (dotted red line).

The Jaccard similarity dendogram of six inflorescence samples indicated presence of an outlier at height > 0.4. The outlier was removed from the present study and the remaining 5 replicates were re-clustered. Biological replicates of each shoot and inflorescence treatment showed absence of outliers at cut-off height = 0.4 (Fig. 1). A multidimensional-scaling (MDS) plot showed apparent dissimilarity between the shoot and inflorescence treatments. Twelve shoot replicates (circles) found on the right panel were distinguished from five inflorescence replicates (crosses) scattered on the left region of dimension 1 at leading logFC = 0 (Fig. 2).

### Gene co-expression network construction and analysis

**Figure 3:**
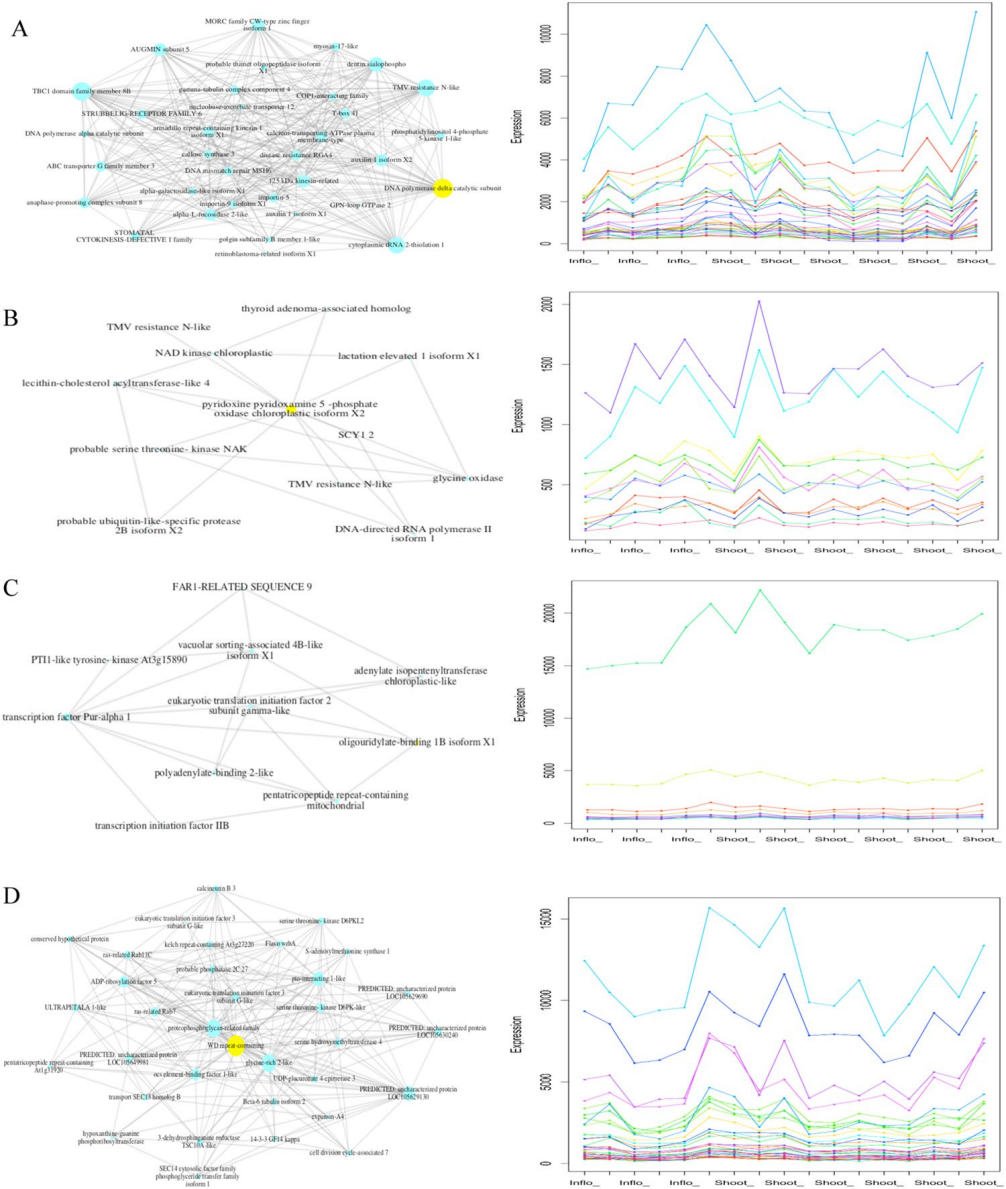
Vicinity networks (VNs) (left) and their corresponding expression profiles (right) extracted based on a gene identifier (yellow node) from *J. curcas* shoot-inflorescence network model. Right: Nodes (blue circles) represent each individual gene. Yellow node in each VCN their corresponding gene identifier: VN1; Jcr4S04537.10, VN2; Jcr4S01867.10, VN3; Jcr4S03942.20 and VN4; Jcr4S03725.10. Edge line (grey) indicates interaction between the nodes. Left: Each coloured line represent a gene and the corresponding expression measures are shown in inflorescence (Inflo_) and shoot (Shoot_) samples (**A–D** represents VN1-VN4).

After filtration, a total of 12 734 genes across 17 samples were applied in the construction of gene co-expression network. Since the transcriptome data utilized in this study showed a non-normal distribution (Supp. 1), the Spearman Correlation Coefficient (SP) was used to establish association between pairs of genes among the samples. Petal’s default function produced a series of thresholds with corresponding mathematical models. We selected the range between the “best” and “alternative’ thresholds (0.845–0.862) to perform a customized refining procedure. A threshold of 0.855 owed the best description for a scale-free architecture and small-world model and was therefore used in all subsequent analyses. The topological properties of the network at a threshold of 0.855 were as follows: Coefficient of determination (R^2^): 0.81, average cluster of co-efficient (meanCC): 0.4146, meanPath: 4.1603, percentage of original dataset utilized in the network model (%used): 96.5%, percentage of vertices that are in the biggest component of the network model (%bigComp): 99.4 (Table 2). The gene co-expression network from 718599 measures contains 12 290 nodes with an average number of neighbors of 117. (Supp. 2). A total of nine vicinity networks (VNs) were obtained from a list of ten gene identifiers. Using a list of gene identifiers^2^, a total of nine VNs were obtained. Number of neighbors and density score for each VN ranged from 10–617 and 0.4–0.63, respectively. Only four VNs (described as VN 1–4) with a density greater than 0.5 were subjected to further characterizations (BLAST description and node’s degree). The node size drawn in each VN indicates their corresponding degree; large node size applies a large degree (Fig. 3).

**Table 2:** Network refined-threshold table.

Threshold	R^2^	slope/power	meanCC	meanPath	%used	%bigComp
0.862	0.82	−1.3305	0.4069	4.3394	95.7515	99.3275
0.86	0.82	−1.2965	0.4099	4.2793	95.9714	99.4027
0.858	0.81	−1.2914	0.4123	4.2296	96.1992	99.4367
0.855	0.81	−1.2877	0.4146	4.1603	96.5133	99.406
0.85	0.80	−1.2481	0.4184	4.0528	96.9609	99.4898
0.845	0.79	−1.2125	0.422	3.9596	97.4242	99.6292

R^2^: Coefficient of determination, meanCC: average cluster coefficient, meanPath: average.

path length, %used: percentage of original dataset utilized in the network model, %bigComp: percentage of vertices that are in the biggest component of the network model.

### Putative involvement of VN1 in epigenetic event, signal transduction and cell wall metabolism

VN1 constructed based on Jcr4S04537.10 (yellow node), described as *DNA polymerase delta catalytic subunit* is depicted in Fig. 3A. It contains 36 nodes connected by 616 edges and the clustering coefficient is 0.766. The average number of neighbors is 16.8 with a density of 0. 51. The sequence IDs and the corresponding descriptions of VN1 genes are given in Supp. Dataset 1. VN1 identified 4 nodes that could be implicated to epigenetic events: *DNA polymerase alpha catalytic subunit*, *DNA polymerase delta catalytic subunit*, *DNA mismatch repair MSH6* and *ABC transporter G family member 3*. DNA polymerases are responsible for the replication of genetic materials, maintenance of genome stability and transmission of genetic information from one generation to the other. Integrity of a genome is constantly affected by endogenous and environmental damaging agents, and thus, a number of DNA repair pathways are constantly recruited for protection. The B family DNA polymerase delta is composed of at least two subunits needed in DNA replication and repair; replisome system, excision repair and recombination repair. The MSH6 gene product forms a complex to recognize base pair mismatches, small insertion or deletions in DNA and subsequently initiate the DNA repair. The mismatch repair is an ATP-driven process^36^. Concomitantly, the ATP consumption in DNA replication and repair was parallel with the presence of *ABC transporter G family member 3* node, which hydrolyzes ATP for transportation of substrate required for DNA repair and RNA translocation^37,38^.

In VN1, several nodes from the repeat protein gene families were identified as follow: *armadillo repeat-containing kinesin 1 isoform X1* node, *MORC family CW-type zinc finger isoform 1* node, and the *TBC1 domain family member 8B* node. Repeat proteins with an ability to bind to peptides and ligands are an integrated component of the protein complexes. Thus, the high binding range of these repeat sequences may facilitate interactions between proteins whilst playing a regulatory role in essential pathways such as plant growth, development and stress tolerance, as previously described in literature^39^. The presence of regulatory nodes in VN1 may also implicate occurrence of the signal transduction, cell division, and floral development processes. The calcium transporting ATPase plasma membrane-type node imparts possible regulation of calcium ions in signal transduction pathway. In Arabidopsis, the stomatal cytokinesis-defective 1–1 (scd1–1), encodes a protein which regulates intracellular protein transport and/or mitogen-activated protein kinase signaling and is also involved in flower morphogenesis^40^. Therefore, the homologue present in VN1 may suggest probable occurrence of floral morphogenesis.

**Figure 4:**
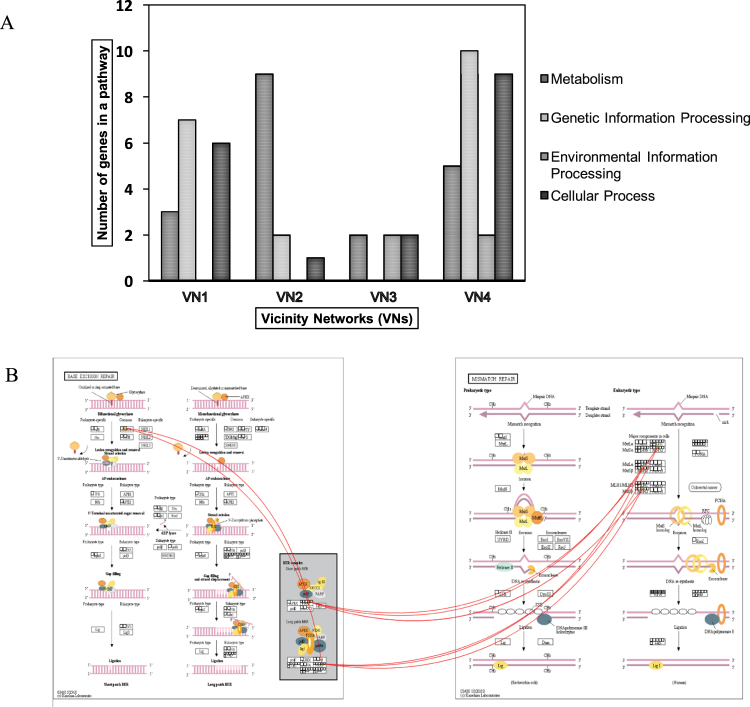
Pathway enrichment analysis: (**A**) Classification of genes in vicinity networks (VNs) into four major maps; metabolism, genetic information processing, environmental information processing and cellular process. (**B**) Correlations (>0.9) between genes in the base excision repair and mismatch repair maps in VN1 are indicated with red curved lines.

Plant cell wall is continually subjected to modification especially during growth and differentiation. In VN1, two nodes namely *Alpha-L-fucosidase 2-like* and the *Alpha-galactosidase-like isoform X1* may have involved in cell wall metabolism. Alpha-L-fucosidase 2-like releases the t-fucosyl residue from xyloglucan oligosaccharides side chain and is a contributing member of the xyloglucan metabolism in Arabidopsis^41^. The xyloglucans are cross-linkers of cellulose microfibers in plant cell wall and thus, impacts the rate of plant cell expansion tremendously. The *galactosidase-like isoform X1* node from the glycosyl hydrolases family are involved in raffinose and galactolipid metabolisms. Cell wall loosening mediates plant cell growth for further expansion^40–42,45^. The presence *Gamma-tubulin complex component 4* node may suggest the re-organization of cytoskeletal microtubules as described in Arabidopsis. In addition, the presence of motor proteins, the *125 kDa kinesin related* and *Myosin 17-like* nodes may have either directly or indirectly affected the movement of the related proteins involved in cell wall metabolism^46^ (Fig. 4A).

### Putative involvement of VN2 in the biosynthesis of chlorophyll molecules and laticifiers

VN2, constructed based on Jcr4S01867.10 (yellow node) described as *pyridoxine pyridoxamine 5-phosphate oxidase chloroplastic isoform* X2 is depicted in Fig. 3B. It contains 12 nodes connected by 11 edges and the clustering coefficient is 0.632. The average number of neighbours is 3.5 with a density of 0.32. The sequence IDs and the corresponding descriptions of VN2 are given in Supp. Dataset 2. In higher plants, the salvage pathway synthesizes nucleotides (purine and pyrimidine) by making use of the intermediates produced by the nucleotide degradative pathway. Bases and nucleotides that were formed during degradation of RNA and DNA are recovered. Pyridoxine (pyridoxamine) of the vitamin B6 group is a potent cofactor for a wide range of biochemical reactions in plants; regulatory molecules in signal transduction and membrane ion transporters, potential antioxidant, facilitates post embryonic root development and confers protection against oxidative stress^47^. The gene identifier (yellow node) and its associated gene members in VN2 may have regulated the formation of laticifer cells and chlorophyll molecules in *Jatropha* shoot system. Among the VN2 neighbours, the *NAD kinase chloroplastic* node may suggest an essential role in chlorophyll synthesis and chloroplast protection against stress. In chloroplast, the NAD kinase catalyzes NAD and ATP into NADP through phosphorylation in presence of sunlight and thus, a number of NADP-dependent biosynthetic pathways are likely to be regulated. For instance, NADPH may have been consumed in reducing reactions in latex biosynthesis. The regulation of NAD kinase activity indicates an increase in metabolic demand which may have occurred due to developmental changes and environmental signals. The presence of *TMV resistance N-like and thyroid adenoma-associated homolog* nodes may infer the occurrence of resistance against stressors, whereas the *lactation elevated 1 isoform X1* may explain possibilities for the biosynthesis of laticifiers, a group of latex containing cells found ubiquitously permeating the *J. curcas* plant body^48^. The presence of *probable serine threonine-kinase NAK* node further explains probable occurrence of signal transduction. The general role of protein kinases is to perceive environmental stimuli from sensor and receptor proteins, process and finally establish the corresponding responses. In line with signal transduction, the *probable ubiquitin-like-specific-protease 2B isoform X2* may have played a role in the developmental process such as activation of stress response and organelle biogenesis. The ubiquitin-specific protease UBP14 affected early embryo development in Arabidopsis^49^ (Fig. 4B).

### Putative involvement of VN3 in heat stress tolerance

VN3, constructed based on Jcr4S03942.20 (yellow node) is depicted in Fig. 3C. It contains 10 nodes connected by 9 edges and the clustering coefficient is 0.67. The average number of neighbors is 4.2 with a density of 0.47. The sequence IDs and the corresponding descriptions of VN3 are given in Supp. Dataset 3. *Jatropha curcas* is a drought and stress tolerant plant. In VN3, the *Oligouridylate-binding 1B isoform X1* node, also a protein component of the stress granules in plants^50,51^ was fixed as the gene identifier. Under heat stress, the mRNA content degrades, however, plant establishes protection via a post-transcriptional process^37^. The mRNA protective mechanism could have incurred in VN3 as evident by the following nodes: *Transcription factor Pur-alpha 1*, *Transcription initiation factor IIB* and *Eukaryotic translation initiation factor 2 subunit gamma-like* and *FAR-RELATED SEQUENCE 9*. In Arabidopsis, FAR1 proteins activates the transcription of light unresponsive genes^52^. The *Polyadenylate-binding 2-like* node may suggest probable stabilization of the nearing degradation mRNAs found within the cell. The primary factor for mRNA degradation is the RNases. They are characterized as relatively non-specific general RNA binding-proteins. On the other hand, the vacuolar RNases make up the bulk of the total RNase activity in plant cells and have been associated to mRNA decay^53^. In VN3, the *Vacuolar-sorting associated 4B-like isoform X1* node may imply occurrence of mRNA degradation while other corresponding neighbors may had established the protection response. The *Pt11-LIKE TYROSINE KINASE Atg15890* node may implicate signal transduction in favor of heat stress. The *Adenylate isopentenyltransferase (IPT) chloroplastic like* node is involved in the cytokinin biosynthetic pathway, where, the isoprenyl group is transferred to form either an adenylate-IPT or tRNA-IPT^54^ (Fig. 3C).

### Putative involvement of VN4 in flowering mechanism

VN4 constructed based on Jcr4S03725.10, the *WD repeat-containing* node (yellow circle) is depicted in Fig. 3D. It contains 34 nodes connected by 33 edges and the clustering coefficient is 0.69. The average number of neighbors is 13.4 with a density of 0.41. The sequence IDs and the corresponding descriptions of VN4 are given in Supp. Dataset 4. The WD repeat-containing complexes are involved in signal transduction, transcriptional regulation^55^ and other plant processes such as meristem organization, cell division and floral development. This module is made of conserved amino acids (44–60) repeat sequence that ends with tryptophan and aspartate (WD) residues^34^. Generally, it serves as rigid scaffolds for protein-protein and protein-DNA interactions^55^. In VN4, the *WD repeat-containing* node, a member of the regulatory protein group which participates in a wide array of biochemical mechanisms and cellular processes^56^ was selected as the gene identifier. There are four predicted uncharacterized proteins and one conserved hypothetical protein in VN4 that could be implicated with probable protein-protein interactions. Other repeat protein nodes present in VN4 are the *Kelch repeat containing At3g27220* and *Pentatricopeptide repeat-containing At1g31920*. The kelch repeat and WD forms a beta-propeller^57^ while the pentatricopeptide repeat encodes the folding of a quadrilateral beta-helix^58^. The repeat families have been characterized in signal transduction, cell division, light signaling, floral development and other transcriptional and signaling mechanisms^59,60^. In addition, the presence of *Ras-related Rab11C* and *Ras-related Rab7* nodes, from the group of small GTPase binding proteins may implicate the event of signal transduction. The GTP-binding proteins are molecular switches that activates and inactivates in the presence of GTP and GDP, respectively^58,60^.

In plants, the shoot apical meristem (SAM) homeostasis enables continuous and reiterative growth of plant organs until termination takes place for the function of floral meristem and flowering. The SAM homeostasis is governed by a feedback loop signaling which involves WUSCHEL (WUS), a homeobox transcription factor encoding stem cell specification at the organizing centre (OC) or the middle region of SAM. The presence of *ULTRAPETALA (ULT) 1-like* node in VN4, a plant specific protein suggests putative regulation of shoot and floral meristem size, floral organ regulation and indeterminate to determinate phase change during *J. curcas* development^61^. In *A. thaliana*, the *ULT1* has been described as a negative regulator of mitotic division taking place in the shoot apical meristems (SAM), where the gene restricts the expression of WUS at OC. A *ULT1* loss-of -function mutants showed enlarged inflorescence and flower meristem size, thereby confirmed the *ULT1* gene function for maintenance of shoot and floral meristem. The *ULT1* is composed of a SAND (Sp100, AIRE-1, NucP41/75 and DEAF-1) putative DNA-interacting domain and a B-box, among others that are actively involved in protein-protein interaction^23,61,62^.

Three nodes associated to protein kinase namely the *serine threonine-kinase D6PK-like*, *serine threonine-kinase D6PKL2* and *serine hydroxymethyltransferase 4* were identified in VN4. Protein kinases are cellular regulatory components of signal transduction in plants. The presence of regulatory nodes such as *protein kinases* and *calcineurin B3* may indicate probable occurrence of cell differentiation, growth and development in the plant system^63^. The calcineurin B like (CBL) proteins are calcium binding proteins that decodes Ca^2+^ signals for signaling and regulatory processes in plants whereas protein serine/threonine kinases have been reported to act as the central processing unit which accepts (environmental conditions) and converts information into appropriate outputs/responses during plant growth and development^64^. A typical plant genome encodes more than 200 putative WDR-containing proteins^39,60,65^. Probable shoot and floral meristem regulations, depicted by the *ULT 1-like* node together with other regulatory nodes further enhanced the role of VN4 in *Jatropha* shoot system homeostasis. Further studies especially mutant analyses of novel proteins in VN4 could potentially confirmed the putative roles of these candidate genes in the production of flowers and inflorescences (Fig. 3D).

### Pathway enrichment analysis

Each VN were described according to the number of genes present in the metabolism, genetic information processing, environmental information processing and cellular process pathways (Fig. 4A). The VN1 showed active participation in genetic information (7 genes) processing; folding sorting and degradation and replication and repair. In addition, the genes under the base excision repair showed strong correlation to mismatch repair map (Fig. 4B). Others include participation in metabolism (3 genes) and cellular processes (6 genes). VN2 was highly involved in metabolism (9); energy (oxidative phosphorylation and nitrogen metabolism), lipid and amino acid. Number of genes in genetic information processing and cell communication were 2 and 1, respectively and genes with putative role in environmental information processing pathway were absent. The genetic information processes include transcription, folding, sorting and degradation and no correlations between the pathway maps were found. Both plant hormone signal transduction and mTOR signaling pathway were recruited under the environmental information processing in VN2. VN3 showed participation in metabolism (amino acid and cofactors and vitamin) environmental information processing and cellular process with 2 genes in each pathway. Environmental processing includes membrane transport and signal transduction. No correlation was found between the pathway maps. Pathway enrichment analysis of VN4 indicated involvement in metabolism (5 genes), genetic (10 genes) and environmental (2 genes) information processing and cellular process (9 genes) (Fig. 4).

### The impact of vicinity networks (VNs) on *J. curcas* yield

Although the *J. curcas* plant has been extensively characterized as a promising biofuel feedstock, an unreliable and inconsistent yield during large-scale cultivation has severely affected the production. Experimental yields ranging from 10–12 tonnes of oil per hectare per year is about 20-fold far exceeding the average yield obtained at field scale; 0.5 tonnes of oil per hectare per year^1–5^. The seed-oil yield is highly dependent on the plant shoot system as the yield index is influenced by various biological processes as followings; vegetative-floral meristem transition, secondary and tertiary branching, floral meristem to floral identity transition, pollen fertility, sex specification and female flower seed setting efficiency^11,12,23^. Nevertheless, the plant shoot system affects the seed yield harvesting index at both vegetative and reproductive states. In plant, the shoot apical meristem (SAM) established during embryogenesis forms all the other above-ground organs and a switch from SAM to inflorescence meristem initiates the plant’s reproductive biology^24,61,62^. Therefore, SAM plays a crucial function in the development of plant shoot system and subsequent seed yield harvesting index. In this study, we report the first gene co-expression network model constructed to study gene-to-gene association among the reproductive-related shoot tissues in *J. curcas* which includes shoot apex bearing SAM, shoot bearing the inflorescence and the inflorescence.

Our vicinity networks (VNs) predicted candidate genes underpinning yield-related biological processes in *J. curcas*. The pathway enrichment analysis of VN1 indicated that a large number of genes were involved in genetic information processes such as folding, sorting and degradation and replication and repair. These events are hallmarks of epigenetic modifications; a genetic process that affects the organism’s phenotype by changing the gene expressions level and chromosome biology without altering the DNA sequence^66^. In several studies conducted across different continents, the occurrence of stable epigenetic events during *J. curcas* development have shown to cause significant epigenetic diversity within and among the accessions^66–69^. On the other hand, others particularly molecular marker studies demonstrated a high phenotypic variation in parallel to low level of genetic diversity^70–73^. These findings suggest that phenotypic variances in *J. curcas* may had been caused by epigenetic polymorphisms as evident by VN1 pathway enrichment analysis. Here, VN1 could be exploited to confirm possible involvement of epigenetics in *J. curcas* development and to understand the effect of epigenetic events on yield-related biological processes.

Belonging to the Euphorbiaceae or the latex bearing family, the *J. curcas* plant is composed of laticifiers or latex producing cells. The articulated (more than one cell is placed on the other) laticifiers contain latex in a suspension emulsion state and are found ubiquitously permeating various tissues in *J. curcas* plant body. Articulate laticifiers originate in both primary and secondary tissues and are composed of predominantly phenolic and others such as starch grains, lipid and mucilage^48^. In VN2, a distinct relationship between the laticifiers and chlorophyll molecule biosynthesis was observed in presence of the vitamin B6 cofactor node. Interestingly, the nodes in VN2 matched the content of latex cells; production of phenolics may have been facilitated by vitamin B6 cofactor whereas starch production by photosynthesis was aided by chlorophyll molecule. The chlorophyll molecules are green pigments that capture light energy during photosynthesis, and the end-product, glucose is either taken up for immediate use by plant or is stored as starch. The *J. curcas* total chlorophyll content had showed positive implication on oil yield^74^. Latex cells indicate production of lipid and concomitantly, lipid metabolism is seen enriched within the VN2. Lipid metabolism is an energy consuming processes, both chlorophyll and latex biosynthesis were found correlated within the VN2 and the result matched with the pathway enrichment of VN2 which indicated highest activity in the metabolism map; energy (oxidative phosphorylation and nitrogen metabolism), lipid and nucleotide.

Despite being a drought-endurable hardy plant, the *J. curcas* suffers from adverse environmental conditions, affecting its productivity tremendously. Heat caused by extreme temperature damages the chlorophyll molecules found on leaves. As a result, a protective response is triggered to encounter or to minimize any further damage that is likely to incur. In parallel with the environmental stress factors, the VN3 was able to explain heats stress mechanisms and potential protective mechanisms which might had taken place in the chlorophyll molecules; the signal transduction and membrane transport processes from the environmental information processes showed the highest activity in the VN3 pathway maps. In addition, the involvement of amino acid metabolism together with cofactors and vitamin metabolism may impart the occurrence protective mechanisms. Therefore, the VN3 may correspond to environmental stresses such as drought and heat conditions.

Seed set and flowering are critical components for seed yield improvement. According to the Arabidopsis flowering model, the LEAFY (LFY) gene plays the central role to regulate flowering; LFY interacts with TERMINAL FLOWER1 (TFL1) and AGAMOUS (AG) for floral signals. In addition, LFY together with APETALA1 (AP1) genes are responsible for the vegetative to reproductive phase switch^75^. Others, with potent role in floral development include the flowering time regulator (SVP) and flowering regulators such as FLOWERING LOCUS D and F, SUPPRESSOR OF OVEREXPRESSION OF CONSTANS1 AND CONSTANS^76^. In a study conducted in China, the JcLFY, an orthologue of the Arabidopsis LFY was overexpressed in *J. curcas* and the subsequent functional analysis significantly affected the inflorescence structure, floral organs and fruit shape^19^. The same study reported expression of LFY transcripts in inflorescence buds, carpels and flower buds (highest). Although numerous findings in seed plants have reported the expression of LFY in inflorescence and flower primordia, our data showed absence of LFY in the *J. curcas* shoot transcriptome data. These results were in agreement with that previously reported in *J. curcas* pedicels and inflorescence. In another study conducted on AP1, a floral meristem and organ identity gene in higher plants, the over-expressed plant showed no apparent effect on the flowering time and floral organs in Jatropha^77^.

Here, VN4 postulated candidate genes that may have involved in *J. curcas* flowering mechanism, although none of these genes have been reported in Arabidopsis flowering model. The presence of ULTRAPETALA (ULT) in VN4 may explain the termination of shoot and floral meristems in *J. curcas*. Under the vegetative state, the shoot apical meristem (SAM) continuously reiterates to produce SAM with the production of leaves and axillary meristems. Flower development is only observed after the differentiation of SAM into floral meristems (FMs)^55^. The pathway analysis of VN4 indicated highest activity in both genetic information processing and cellular process. Cellular processes which include transport and catabolism, cell growth and death and cell communication further corroborated the putative role of VN4 in tissue differentiation. Therefore, the VN4 may be employed in flowering enhancement strategies such improved rate of SAM to inflorescence and/or floral meristem switch and increased number of inflorescence in *J. curcas* plant.

## Conclusion

This is the first gene expression profile-based gene co-expression network that investigated the gene-to-gene associations in *J. curcas* reproductive-related shoot system. An integration of present results and previous data available in literature elucidated the putative gene functions of vicinity networks involved in key biological processes associated to yield. We presented possible models underpinning the biosynthesis of chlorophyll molecules and laticifier cells, cell wall metabolism, flowering, heat stress tolerance and epigenetic events during *J. curcas* growth and development. The putative candidate genes modelled in the vicinity networks are of great importance in breeding strategies and subsequent production of superior and efficient *J. curcas* varieties. However, the candidate genes presented in each VN along with their expression profile requires functional validation prior to downstream application in *J. curcas* breeding programs.

## Electronic supplementary material

Supplementary Info 1

Supplementary Info 2

Dataset 1

Dataset 2

Dataset 3

Dataset 4

## References

[1] Dias, L. A., Missio, R. F. & Dias, D. C. Antiquity, botany, origin and domestication of *Jatropha curcas Euphorbiaceae*), a plant species with potential for biodiesel production. *Genetics and Molecular Research* 2719–2728, 10.4238/2012.June.25.6 (2012). 10.4238/2012.June.25.6 22782638

[2] Fairless D (2007). Biofuel: the little shrub that could-maybe. Nature.

[3] Ghosh A (2007). Prospects for Jatropha methyl ester (biodiesel) in India. International Journal of Environmental Studies.

[4] Jain S, Sharma MP (2010). Biodiesel production from *Jatropha curcas* oil. Renewable & Sustainable Energy Reviews.

[5] Koh MY, Idaty T, Ghazi M (2011). A review of biodiesel production from *Jatropha curcas* L. oil. Renewable & Sustainable Energy Reviews.

[6] Mandpe S, Kadlaskar S, Degen W, Keppeler S (2005). On road testing of advanced common rail diesel vehicles with biodiesel from the *Jatropha curcas* plant. Soc. Automot. Eng..

[7] Openshaw. A review of *Jatropha curca*s: an oil plant of unfulfilled promise. *Biomass Bioenergy* **9**, 1–15 10.1016/S0961-9534(00)00019-2 (2000).

[8] Prakash AR, Singh S, Prakash CR, Ghosh A, Agarwal PK (2016). Development of Jatropha hybrids with enhanced growth, yield and oil attributes suitable for semi-arid wastelands. Agroforestry Systems.

[9] Alvarez-Buylla ER (2010). Flower development. Arabidopsis.

[10] Schmitz G, Theres K (2005). Shoot and inflorescence branching. Current Opinion in Plant Biology.

[11] Bhattacharya A, Kalayani D, Subodh KD (2005). Floral biology, floral resource constraints and pollination limitation in *Jatropha curcas* L. Pakistan J Biol Sci.

[12] Bhuva, H. *et al*. Variability in growthand yield parameters in different provenances of *Jatropha curcas* on marginal land of Gujarat. In: Satish Kumar M (ed.) Bio-fuel plants, pp 139–146 (2007).

[13] Azevedo Peixoto LD, Laviola BG, Alves AA, Rosado TB, Bhering LL (2017). Breeding *Jatropha curcas* by genomic selection: A pilot assessment of the accuracy of predictive models. PLoS ONE.

[14] Costa GGL (2010). Transcriptome analysis of the oil-rich seed of the bioenergy crop *Jatropha curcas* L. BMC Genomics.

[15] Dhakshanamoorthy D, Selvaraj R, Chidambaram AL (2011). Induced mutagenesis in *Jatropha curcas* L. using gamma rays and detection of DNA polymorphism through RAPD marker. *Comptes Rendus*. Biology.

[16] Gangwar M, Sood H, Chauhan RS (2016). Genomics and relative expression analysis identifies key genes associated with high female to male flower ratio in *Jatropha curcas* L. Molecular Biology Reports.

[17] King AJ, Li Y, Graham IA (2011). Profiling the Developing *Jatropha curcas* L. Seed Transcriptome by Pyrosequencing. *Bioenergy*. Research.

[18] Maghuly F, Laimer M (2013). *Jatropha curcas*, a biofuel crop: Functional genomics fr understanding metabolic pathways and genetic improvement. Biotechnology Journal.

[19] Tang M (2016). An ortholog of *LEAFY* in *Jatropha curcas* regulates flowering time and floral organ development. Scientific Reports.

[20] Xu G, Huang J, Yang Y, Yao Y (2016). Transcriptome analysis of flower sex differentiation in *Jatropha curca*s L. using RNA Sequencing. PLoS ONE.

[21] Che-Mat NH, Bhuiyan MAR, Senan S, Yaakob Z, Ratnam W (2015). Selection of high yielding *Jatropha curcas* L. accessions for elite hybrid seed production. Sains Malaysiana.

[22] Barton MK, Poethig RS (1993). Formation of the shoot apical meristem in *Arabidopsis thaliana*: an analysis of development in the wild type and in the *shoot meristemless* mutant. Development.

[23] Benlloch R, Berbel A, Serrano-Mislata A, Madueno F (2007). Floral initiation and inflorescence architecture: A comparative view. Ann. Bot..

[24] Poethig RS (1990). Phase change and the regulation of shoot morphogenesis in plants. Science.

[25] Petereit J, Smith S, F. C. H, Schlauch KA (2016). petal: Co-expression network modelling in R. BMC Systems Biology.

[26] Schaefer RJ, Michno JM, Myers CL (2016). Unraveling gene function in agricultural species using gene co-expression networks. Biochimica et Biophysica Acta - Gene Regulatory Mechanisms.

[27] Allocco DJ, Kohane IS, Butte AJ (2004). Quantifying the relationship between co-expression, co-regulation and gene function. BMC Bioinformatics.

[28] Ouyang Y, Huang X, Lu Z, Yao J (2012). Genomic survey, expression profile and co-expression network analysis of OsWD40 family in rice. BMC Genomics.

[29] Usadel B (2009). Co-expression tools for plant biology: opportunities for hypothesis generation and caveats. Plant Cell Environment.

[30] Govender N, Senan S, Mohamed-Hussein ZA, Ratnam W (2017). Genomics Data Transcriptome analysis of reproductive tissue differentiation in *Jatropha curcas* L. *Genomics*. Data.

[31] Sangha JS, Gu K, Kaur J, Yin Z (2010). An improved method for RNA isolation and cDNA library construction from immature seeds of *Jatropha curcas* L. BMC Research Notes.

[32] Conesa A (2005). Blast2GO: a universal tool for annotation, visualization and analysis in functional genomics research. Bioinformatics.

[33] Gotz S (2008). High-throughput functional annotation and data mining with the Blast2GO suite. Nucleic Acids Research.

[34] Neer EJ, Schmidt CJ, Nambudripad R, Smith TF (1994). The ancient regulatory-protein family of WD-repeat proteins. Nature.

[35] Sakurai N (2011). KaPPA-View4: a metabolic pathway database for representation and analysis of correlation networks of gene co-expression and metabolite co-accumulation and omics data. Nucleic Acids Research.

[36] Antony E, Hingorani MM (2003). Mismatch recognition-coupled stabilization of Msh2-Msh6 in an ATP-bound state at the initiation of DNA repair. Biochemistry.

[37] Chau, N. C. *et al*. Oligouridylate binding protein 1b plays an integral role in plant heat stress tolerance. *Frontiers in Plant Science*, 10.3389/fpls.2016.00853 (2016). 10.3389/fpls.2016.00853 PMC4911357 27379136

[38] Davidson AL, Dassa E, Orelle C, Chen J (2008). Structure, function, and evolution of bacterial ATP-binding cassette systems. Microbiology & Molecular Biology Reviews.

[39] Sharma M, Pandey GK (2015). Expansion and function of repeat domains proteins during stress and development in plants. Frontiers in Plant Science.

[40] Falbel TG (2003). SCD1 is required for cell cytokinesis and polarized cell expansion in *Arabidopsis thaliana*. Development.

[41] Kurth EG (2017). Myosin-driven transport network in plants. PNAS.

[42] Long X, He B, Fang Y, Tang C (2016). Identification and characterization of the glucose phosphate dehydrogenase gene family in the para rubber tree, *Hevea brasiliensis*. Frontiers in Plant Science.

[43] Mangeon A, Junqueira RM, Sachetto-martins G (2010). Functional diversity of the plant glycine-rich proteins superfamily. Plant signaling & behavior.

[44] Marowa P, Ding A, Kong Y (2016). Expansins: roles in plant growth and potential applications in crop improvement. Plant Cell Reports.

[45] Tenhaken R (2015). Cell wall remodeling under abiotic stress. Frontiers in Plant Science.

[46] Turner WL, Waller JC, Snedden WA (2004). Identification, molecular cloning and functional characterization of a novel NADH kinase from Arabidopsis thaliana. Biochemical Journal.

[47] Martino, L., Safo, M. K., Musayev, F. N., Bossa, F. & Schirch, V. Structure and mechanism of *Escherichia col*i pyridoxine 5 V -phosphate oxidase. **1647**, 76–82 (2003). 10.1016/s1570-9639(03)00060-8 12686112

[48] Krishnamurthy *et al*. Chapter 1: Laticifers of Jatropha. Bahadur B. *et al*. (eds), In Jatropha, Challenges for a new energy crop: Vol 2: Genetic improvement and Biotechnology, 10.1007/978-1-4614-4915-7_1 2013).

[49] Doelling JH, Yan N, Kurepa J, Walker J, Vierstra RD (2001). The ubiquitin-specific protease UBP14 is essential for early embryo development in *Arabidopsis thaliana*. Plant Journal.

[50] Lambermon MH (2000). UBP1, a novel hnRNP-like protein that functions at multiple steps of higher plant nuclear pre-mRNA maturation. EMBO Journal.

[51] Weber C, Nover L, Fauth M (2008). Plant stress granules and mRNA processing bodies are distinct from heat stress granules. Plant Journal.

[52] Hudson ME, Lisch DR, Quail PH (2003). The *FHY3* and *FAR1* genes encode transposase-related proteins involved in regulation of gene expression by the phytochrome A-signaling pathway. The Plant Journal..

[53] Green PJ (1993). Control of mRNA stability in higher plants. Plant Physiology.

[54] Lindner AC (2014). Isopentenyltransferase-1 (IPT1) knockoutin *Physcomitrella* together with phylogenetic analyses of IPTs provide insights into evolution of plant cytokinin biosynthesis. Journal of Experimental Botany..

[55] Miller JC, Chezem WR, Clay NK (2016). Ternary WD40 repeat-containing protein complexes: Evolution, composition and roles in plant immunity. Frontiers in Plant Science.

[56] van Nocker, S., & Ludwig, P. (2003). The WD-repeat protein super family in Arabidopsis: conservation and divergence in structure and function. *BMC Genomics* **4**, 10.1186/1471-2164-4-50 (2003). 10.1186/1471-2164-4-50 PMC317288 14672542

[57] Adams J, Kelso R, Cooley L (2000). The kelch repeat superfamily of proteins: propellers of cell function. Trends Cell Biol..

[58] Vetting MW (2006). Pentapeptide repeat proteins. Biochemistry.

[59] Saito R (2012). A travel guide to Cytoscape plugins. Nature Methods.

[60] Villanueva MA, Islas-flores T, Ullah H (2016). Editorial: Signaling through WD-Repeat Proteins inPlants. Frontiers in Plant Science.

[61] Moreau F (2016). The Myb-domain protein ULTRAPETALA1 INTERACTING FACTOR 1 controls floral meristem activities in Arabidopsis. Development.

[62] Coen ES (1990). FLORICAULA a homeotic gene required for flower development in *Antirrhinum-majus*. Cell.

[63] Stress B (2015). Functional roles of plant protein kinases in signal transduction pathways during sbiotic and biotic stress. Journal of Biodiversity, Bioprospecting and Development.

[64] Hardie DG (1999). PLANT PROTEIN SERINE/THREONINE KINASES: Classification and functions. Annual Review of Plant Physiology and Plant Molecular Biology.

[65] Mesarich CH, Bowen JK, Hamiaux C, Templeton MD (2015). Repeat-containing protein effectors of plant-associated organisms. Frontiers in Plant Science.

[66] Yi, C., Zhang, S., Liu, X., Bui, H. T. N. & Hong, Y. Does epigenetic polymorphism contribute to phenotypic variances in *Jatropha curcas* L? *BMC Biology*10.1186/1471-2229-10-259 (2010). 10.1186/1471-2229-10-259 PMC3017842 21092236

[67] Li H (2017). Genetic tracing of *Jatropha curcas* L. from its mesoamerican origin to the worldfront. Frontiers in Plant Science.

[68] Montes Osorio, L. R. *et al*. High level of molecular and phenotypic biodiversity in *Jatropha curcas* from Central America compared to Africa, Asia and South America. *BMC Plant Biology* **14**, 10.1186/1471-2229-14-77 (2014). 10.1186/1471-2229-14-77 PMC3987055 24666927

[69] Popluechai, S. *et al*. Narrow genetic and apparent phenetic diversity in *Jatropha curcas*: initial success with generating low phorbol ester interspecific hybrids. *Nature Proceedings* (2009).

[70] Senan S, Ismail I, Ratnam W (2016). Genetic homogeneity in *Jatropha curcas* L. individuals as revealed by microsatellite markers: Implication to breeding strategies. Brazillian Journal of Botany.

[71] Shabanimofrad M, Rafii MY, Megat W, Biabani AR, Latif MA (2013). Phenotypic, genotypic and genetic divergence found in 48 newly collected Malaysian accessions of *Jatropha curcas* L. Industrial Crops and Products..

[72] Sudheer DVNP, Mastan SG, Rahman H, Reddy MP (2010). Molecular characterization and genetic diversity analysis of *Jatropha curcas* L. in India using RAPD and AFLP analysis. Molecular Biology Reports.

[73] Yue GH (2013). No variation at 29 microsatellites in the genome of *Jatropha curcas*. Journal of Genomics.

[74] Singh S (2016). Trait selection by path and principal component analysis in *Jatropha curcas* for enhanced oil yield. Industrial Crops and Products.

[75] Weigel D, Alvarez J, Smyth DR, Yanofsky MF, Meyerowitz EM (1992). LEAFY controls floral meristem identity in *Arabidopsis*. Cell.

[76] Liljegren SJ, Gustafson-Brown C, Pinyopich A, Ditta GS, Yanofsky MF (1999). Interactions among APETALA1, LEAFY, and TERMINAL FLOWER1 specify meristem fate. Plant Cell.

[77] Torre, F., De, Sampedro, J., Zarra, I. & Revilla, G. AtFXG1, an Arabidopsis gene Encoding L-fucosidase active against fucosylated xyloglucan oligosaccharides 1. **128**, 247–255, 10.1104/pp.010508 (2002). PMC148987 11788770

