# Supplementary material for: A gene co-expression network model identifies yield-related vicinity networks in Jatropha curcas shoot system
Source: Sci Rep. 2018 Jun 15;8:9211. doi: 10.1038/s41598-018-27493-z (PMC6003958; doi:10.1038/s41598-018-27493-z)
Supplement: Supplementary file 1 — Supplementary Info 1 [file 41598_2018_27493_MOESM1_ESM.pdf]

A gene co-expression network model identifies yield-related vicinity networks in *Jatropha curcas* shoot system

Nisha Govender[1, 2]*, Siju Senan[1], Zeti-Azura Mohamed-Hussein[2, 3], Wickneswari Ratnam[1]
[1]School of Environmental and Natural Resource Sciences, Faculty of Science and Technology, Universiti Kebangsaan Malaysia, 43600 UKM Bangi, Selangor, Malaysia.
[2] Center for Bioinformatics Research, Institute of Systems Biology (INBIOSIS), Universiti Kebangsaan Malaysia, 43600 UKM Bangi, Selangor, Malaysia.
[3] School of Biosciences and Biotechnology, Faculty of Science and Technology, Universiti Kebangsaan Malaysia, 43600 UKM Bangi, Selangor, Malaysia.

*Corresponding author: nishag@ukm.edu.my

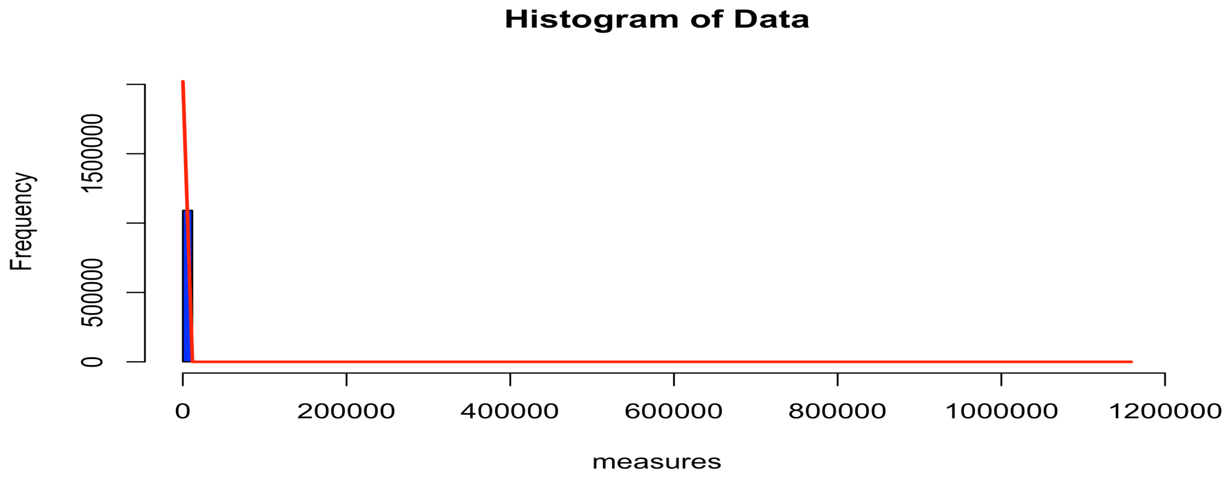

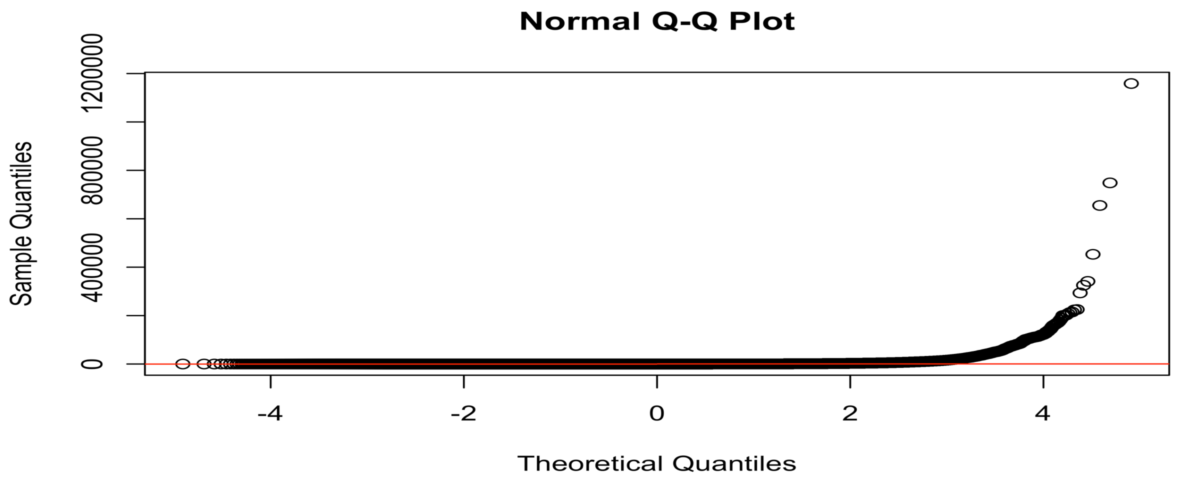

Supp. 1: Histogram (top) and Normal Q-Q Plot (bottom) of transcriptome count data distribution. The data is generated from 5 inflorescence and 12 shoot samples of *Jatropha curcas*.
