# Supplementary material for: A gene co-expression network model identifies yield-related vicinity networks in Jatropha curcas shoot system
Source: Sci Rep. 2018 Jun 15;8:9211. doi: 10.1038/s41598-018-27493-z (PMC6003958; doi:10.1038/s41598-018-27493-z)
Supplement: Supplementary file 2 — Supplementary Info 2 [file 41598_2018_27493_MOESM2_ESM.pdf]

A gene co-expression network model identifies yield-related vicinity networks in *Jatropha curcas* shoot system

Nisha Govender[1, 2]*, Siju Senan[1], Zeti-Azura Mohamed-Hussein[2, 3], Wickneswari Ratnam[1]
[1]School of Environmental and Natural Resource Sciences, Faculty of Science and Technology, Universiti Kebangsaan Malaysia, 43600 UKM Bangi, Selangor, Malaysia.
[2] Center for Bioinformatics Research, Institute of Systems Biology (INBIOSIS), Universiti Kebangsaan Malaysia, 43600 UKM Bangi, Selangor, Malaysia.
[3] School of Biosciences and Biotechnology, Faculty of Science and Technology, Universiti Kebangsaan Malaysia, 43600 UKM Bangi, Selangor, Malaysia.

*Corresponding author: nishag@ukm.edu.my

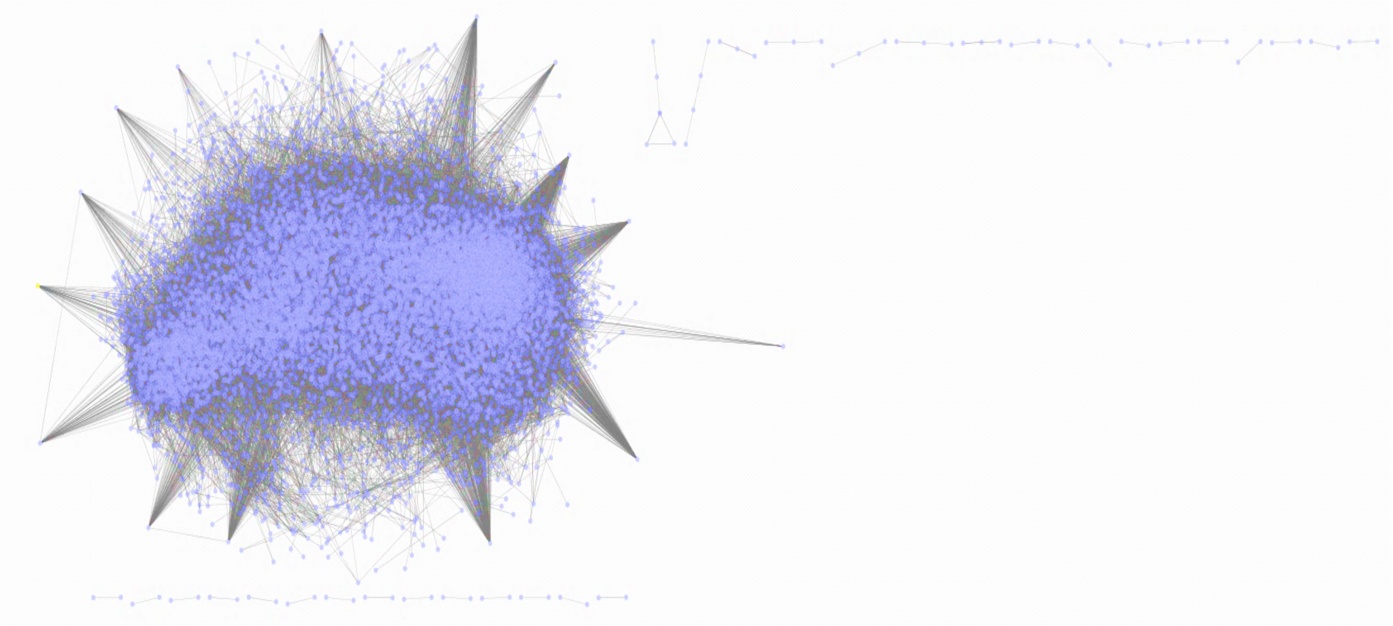

Supp. 2: A gene-co-expression network model built from transcriptome data depicts the *Jatropha curcas* inflorescence and shoot molecular interactions. The network model is visualized on Cytoscape with a spring embedded layout application. Nodes (purple circles) represent each individual gene. Edge line (grey) indicates interaction between the nodes. The network model contains 12 290 number of nodes with 117 average number of neighbors.
